# Raising the Topic: Clinical Needs Assessment and Co‐Design of Targeted Clinical Resources for Primary Healthcare Practitioners to Prevent and Manage Childhood Obesity

**DOI:** 10.1002/hpja.70033

**Published:** 2025-03-13

**Authors:** Oliver J. Canfell, Jacqueline Cotugno, Joanna Munro, Leanna Woods, Robyn Littlewood, Jacqueline L. Walker

**Affiliations:** ^1^ Department of Nutritional Sciences Faculty of Life Sciences and Medicine, King's College London London UK; ^2^ Health and Wellbeing Queensland Queensland Government, the State of Queensland Brisbane Australia; ^3^ Queensland Digital Health Centre Centre for Health Services Research, Faculty of Medicine, the University of Queensland Herston Australia; ^4^ School of Human Movement and Nutrition Sciences Faculty of Health and Behavioural Sciences, the University of Queensland St Lucia Australia

**Keywords:** childhood obesity, co‐design, healthcare professionals, preventive medicine, primary health care, risk factors

## Abstract

**Introduction:**

This study aimed to (a) determine the unmet clinical resource needs of multidisciplinary primary healthcare practitioners (PHPs) to overcome evidence‐based barriers to preventing and managing childhood obesity in practice; and (b) co‐design precision solutions to the identified needs of PHPs.

**Methods:**

This qualitative study was conducted across three phases: (1) clinical needs assessment with 18 multidisciplinary PHPs over five virtual focus groups, (2) participatory, user‐centred co‐design via an online design workshop with five PHPs and four caregivers, and (3) clinical resource prototype development and qualitative user feedback. Data was thematically analysed using the Framework Method.

**Results:**

Clinical needs assessment (phase 1) identified unmet resource needs across four themes: (a) visual and simple clinically integrated media; (b) positive, health‐focused language and countering shame; (c) referral opportunities and clinical upskilling in priority areas; (d) practical family‐based, culturally appropriate and early years focus. Co‐design (phase 2) developed nine clinical resource solutions. A prototype was developed for clinical piloting that targets the pervasive barrier of initiating a conversation about weight and healthy growth—the *BRAVE* (*Build relationships, Raise respectfully, Ask about attitudes, Validate values, Engage & enable*) framework. The purpose of *BRAVE* is to improve PHP self‐efficacy to raise the topic of weight and to provide a safe, trusted and empowering environment for children and families.

**Conclusions:**

This study uncovered unmet clinical resource needs for PHPs to confidently address childhood obesity and co‐designed a new clinical resource to help PHPs raise the topic of weight with families that is ready for clinical piloting.

## 1 Introduction

Childhood overweight and obesity (overweight/obesity) is a significant national and global health burden. In Australia, more than one in four (27.7%) children and adolescents (aged 5–17 years) live with overweight or obesity [1]. Addressing childhood overweight/obesity at population scale is a national priority: the previously launched *National Obesity Strategy* (March 2022) has committed to reducing childhood overweight/obesity prevalence by at least 5% by 2030 [2]. Treatment is difficult as clinical obesity is a chronic, relapsing and progressive disease process [3, 4]. Prevention of preclinical and clinical obesity requires prioritisation in practice [4] however, treatment and management remain the prevailing model of care.

Primary health care (PHC) has been confirmed as a critical setting for childhood overweight/obesity prevention and treatment [5]. Despite this, most children living with overweight/obesity do not receive evidence‐based care in PHC [6]. In Australia, for every 200 children who visit their general practitioner (GP) in PHC, 60 live with overweight/obesity, but only one is offered weight management support [7]. Evidence has identified multicomponent barriers to managing childhood overweight/obesity in PHC, including perceived self‐efficacy, weight stigma and difficulty raising the topic of weight [5]. The focus on barriers to date has been effective in highlighting the problem; however, solutions must be prioritised to promote action. Previously in Australia, a call was made by clinician‐researchers to develop accessible resources, pathways, and training in childhood obesity for PHPs to use in conjunction with caregivers [8]. Clinical resources and tools for the management of childhood overweight/obesity exist [9, 10], such as those available in the Australian *Healthy Kids for Professionals* [11] online portal and *Clinicians Hub* [12]; however, gaps remain in targeted resources to help PHPs overcome pervasive evidence‐based barriers in practice.

There are two research gaps that we sought to address: (1) understanding the clinical needs of PHPs to address childhood obesity at the point‐of‐care and (2) understanding how clinical resources could be co‐designed that precisely target evidence‐based barriers to preventing and managing childhood obesity at the point‐of‐care. To address these gaps, a participatory, user‐centred co‐design a method was adopted to promote empowerment, ownership and sustainable uptake of any designed resources or interventions [13, 14]. Therefore, our research questions were:

1. What are the unmet clinical resource needs of PHPs to overcome evidence‐based barriers to preventing and managing childhood obesity in practice?
2. How can we co‐design targeted clinical resources with PHPs and caregivers to address the identified needs of PHPs?

We hypothesised that PHPs would identify unmet clinical resource needs in priority areas of raising the topic of weight, priority population groups (First‐Nations people, culturally and linguistically diverse people), behaviour change strategies and communicating sensitively about weight. Our study aimed to answer each research question by conducting a clinical needs assessment with PHPs (research question 1) and participatory, user‐centred co‐design with PHPs and caregivers (research question 2) to co‐develop resources ready for clinical piloting.

## 2 Methods

### 2.1 Design

**FIGURE 1:**
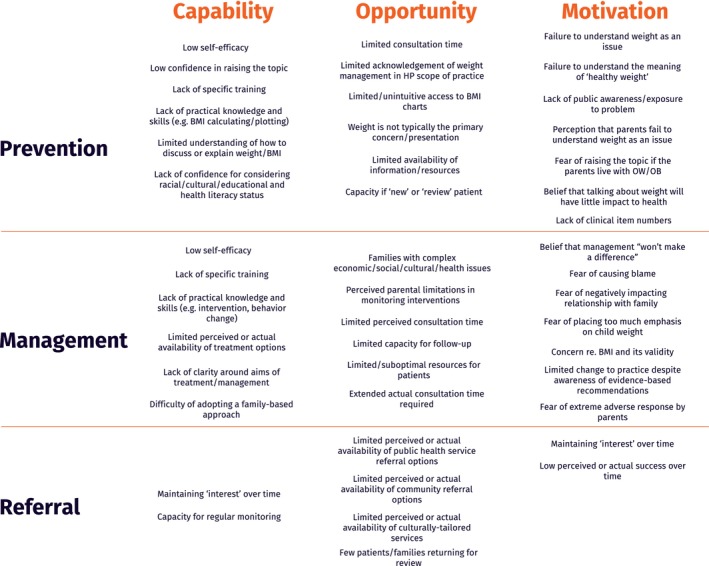
Evidence map of the clinical barriers to prevention, management and referral of childhood overweight/obesity in primary health care.

A qualitative cross‐sectional study was conducted in three phases in alignment with the research aims, i.e., phase one involved a clinical needs assessment and phases two–three involved participatory, user‐centred co‐design. In phase one, we used the Capacity, Opportunity, Motivation—Behaviour (COM‐B) behaviour change wheel [15] to create an evidence map (Figure 1) of barriers to the prevention, management and referral for childhood obesity in practice. The evidence map was then used to inform an evidence‐based clinical needs assessment with PHPs [16]. In phase two, principles of participatory and user‐centred design [13, 17, 18] were adopted with PHPs and caregivers to co‐design solutions to the clinical needs identified in phase one. Phase two incorporated three participatory activities in a collaborative design workshop: (1) individual visual design sketch, (2) Nominal group strategy, (3) Storyboarding. Phase three comprised prototype development of a novel precision clinical resource for childhood overweight/obesity and sought participant feedback. Co‐design and participatory methods seek to actively involve all stakeholders in a process to understand the lived experiences, needs, behaviours and preferences of users [19]. The exploratory and collaborative process helps develop tailored solutions that are relevant to the context and accepted by end‐users [19]. The Consolidated criteria for Reporting Qualitative research (COREQ) checklist was adhered to [20] (File S1). This research was approved by an institutional human research ethics committee (approval number blinded).

### 2.2 Participants and Recruitment

PHPs and caregivers were recruited from across a large state in Australia. PHPs were eligible if they engaged in the clinical management of children or young people with overweight/obesity, including: General Practitioner (GP), Child Health Nurse, General Practice Nurse, Community Nurse, Midwives and Allied Health Professionals (e.g., Accredited Practising Dietitian, Psychologist, Clinical Exercise Physiologist, Multicultural Health Worker and Aboriginal and Torres Strait Islander Health Workers). Caregivers were defined as the parent or legal guardian of a child or young person (aged 0–17 years).

Participants (PHPs and caregivers) were recruited across diverse networks of health services and local communities in a large state in Australia, using existing clinical and consumer email networks and digital media. Potential participants expressed interest via email and could then provide consent online before completing a short demographics questionnaire. Participants who expressed initial interest but did not engage further were followed up via two emails over a 2‐week period. Non‐response was classified as no communication following the expression of interest. No interviewer‐participant relationship existed prior to the study commencement. Participant knowledge of the interviewer (author initials) was limited to the information provided in the information sheet. Participants were offered a $20 gift card as reimbursement for participation in the study.

### 2.3 Data Collection

#### 2.3.1 Participant Demographics

Demographic data were collected via a secure online survey tool—*Checkbox* (v7.6). PHPs were asked to report their clinical discipline, health service affiliations and years practising as clinicians. Caregivers were asked to report the number and age of their children, and which PHP disciplines they had previously seen for the care of their child.

#### 2.3.2 Phase One—Clinical Needs Assessment

PHPs were invited to participate in one focus group. The number of focus groups needed to identify 80%–90% of thematic codes across a study is 3–6 [21]. Our target number of focus groups was five (*n* = 3–4 participants per group) and aimed to purposively distribute PHPs across each focus group to ensure multidisciplinary perspectives were represented. Prior to commencing the focus group protocol, the lead facilitator (author initials) delivered a short (10 min) presentation on evidence‐based barriers to the prevention, management and referral of childhood overweight/obesity in practice. Focus group questions (Table 1) were semi‐structured and subdivided into lead questions and probing questions related to study aim 1. Previous literature and the theoretical foundation of the current study were used to guide question design [16, 22]. Focus groups were audio recorded and transcribed verbatim by an external transcription service. No repeat focus groups were conducted. Transcribed text was not returned to participants unless requested.

**TABLE 1:** Semi‐structured focus group protocol for PHPs—Clinical Needs Assessment (*n* = 18).

Questions	Prompts
Considering the evidence‐based barriers to preventing and managing childhood obesity in practice:	
What are the current gaps in clinical resources for childhood obesity?	For: Raising the topic of weight? Identifying obesity? Preventing obesity? Treating and managing obesity? Referrals related to obesity?
How could new resources be designed based on child age?	For: Infancy and early childhood (i.e., first 2000 days)? Middle‐late childhood (i.e., 6–11 years)? Adolescence (i.e., 12–17 years)?
What considerations need to be made for priority populations?	For: Culturally and linguistically diverse people? People living with socioeconomic disadvantage?
How could new resources best be integrated in routine clinical practice?	Nil
What would be the most important enablers to using new resources?	Nil
What would be the most important barriers to using new resources?	Nil

#### 2.3.3 Phase Two—Participatory, User‐Centred Co‐Design

PHPs and caregivers were invited to participate in one facilitated co‐design workshop (1.5 h duration) using an online collaborative whiteboard—*Miro*. Our sample target was 8–10 participants to ensure diverse representation of PHP and caregiver demographics. One workshop was conducted to ensure design activities were sequenced appropriately and to maximise the richness of idea generation. All PHP participants who participated in phase one (clinical needs assessment) were invited to phase two (participatory, user‐centred co‐design). Results of phase one were used to inform key components of the design workshop, including the goal, requirements of any co‐designed solution and workshop scope (Table 2). Before commencing the workshop, the lead facilitator (JC) delivered a short (10 min) presentation on (a) evidence‐based barriers to preventing and managing childhood obesity in practice and (b) thematic results of phase one to provide PHP‐identified unmet clinical resource needs.

**TABLE 2:** Key components of the design workshop—participatory, user‐centred co‐design.

Goal	To build solutions for PHPs to routinely talk to and help families with growth, weight and health behaviours
Requirements	Help PHPs talk about weight with families Help PHPs deliver messages in a simple, practical and family‐centred way Be easily integrated within current systems and clinical workflow
Scope	
In scope	Resources for PHPs Prevention and management of childhood overweight and obesity
Out of scope	Consumer (e.g., parent/guardian) resources
	School‐based resources

Abbreviation: PHPs, primary healthcare professionals.

Participants were asked to complete three activities—one individual and two group activities using *Miro*:

1. An individual visual design “sketch”—using sticky notes, icons and emojis—to storyboard a potential solution that addressed the workshop goal, requirements and scope. Participants were then invited to present their sketch to the group.
2. Nominal group strategy—participants used love heart emojis to vote for their favourite solutions (not including their own), followed by a group discussion to reach consensus on the leading solutions.
3. Storyboarding—Participants Were Tasked With Using the Leading Solutions to Tell a Visual Story of How the Problem (Unmet PHP Clinical Resource Needs) Could Be Solved, Including Accompanying Text That Described “what is happening here”? A Single Solution or Multiple (Integrated) Solutions Could Be Applied to the Storyboard

#### 2.3.4 Phase Three—Prototype Development and Participant Feedback

A prototype solution was developed following phase two. All phase two participants were sent the prototype clinical resource via email and invited to provide informal qualitative (written) feedback on four questions: (1) *what do you like about the solution?* (2) *what do not you like?* (3) *what could be improved?* (4) *how should the prototype be communicated?* The prototype was then refined according to participant feedback.

### 2.4 Data Analysis

#### 2.4.1 Phase One—Clinical Needs Assessment

Data analysis was conducted using a modified, rapid version of the Framework Method for qualitative data analysis [23]. The modified version used an inductive nominal group technique to synthesise, interpret and reach consensus on final themes. One transcript was allocated to one investigator. Each investigator performed line‐by‐line coding and generated high‐level preliminary themes. Researchers then performed a rapid thematic analysis using a virtual whiteboard and a ‘cluster and name’ technique to generate a working analytical framework. Researchers presented their code and preliminary themes to the group, which were transcribed onto the virtual whiteboard. Through an iterative and interpretive process, researchers then grouped similar concepts into parent themes. Discrepancies were resolved with discussion until the final themes were decided and approved by consensus.

#### 2.4.2 Phase Two—Participatory, User‐Centred Co‐Design

Data analysis was conducted by consolidating (a) solutions with the highest number of participant votes with (b) the final storyboard. Following the workshop, the research team held internal discussions to ensure the workshop outputs were suitable clinical resources to develop as a prototype. The solution of most relevance to the research question was prioritised, and a low‐fidelity, text‐based prototype representing this solution was generated.

#### 2.4.3 Phase Three—Prototype Development and Participant Feedback

Qualitative participant feedback to the clinical resource prototype was reviewed in research team meetings and the prototype was updated according to responses to the questions ‘what don't you like?’ and ‘what could be improved’?

## 3 Results

### 3.1 Study Population

A total of 33 PHPs and 9 caregivers initially expressed interest in participating in the study. Of these, 18 PHPs and 4 caregivers consented to participate. File S2 summarises participant characteristics for phase one (clinical needs assessment) and phase two (participatory, user‐centred co‐design). The primary reason for attrition following the initial expression of interest was non‐response to follow‐up communications.

#### 3.1.1 Phase One—Clinical Needs Assessment

A total of 18 PHPs participated in five focus groups (*n* = 3–4 per focus group; duration = 67–86 min [min‐max]). Most PHPs represented either nursing (8, 44%) or allied health (7, 39%) professions and included a diverse mix of disciplines, such as child health nurses (3, 17%), dietitians (3, 17%) and clinical exercise physiologists (2, 11%). Of those who reported an affiliation with public health services in our large state in Australia, most (8, 62%) were from major cities, whilst the remainder represented inner regional (1, 8%), outer regional (2, 15%) or very remote (2, 15%) areas. PHPs were highly experienced, with 15 (83%) reporting > 11 years practising as a clinician.

#### 3.1.2 Phase Two—Participatory, User‐Centred Co‐Design

All PHPs (*n* 18) from phase one were invited to participate in phase two. A total of five PHPs (*n* 13 declined) and four caregivers agreed to participate (total *n* 9). PHP participants were diverse across disciplines and geographical areas, with nursing, allied health and medical representation from metropolitan, regional and remote areas in the large Australian state. Caregivers were parents/guardians of children aged 6–17 years and had previously seen child health nurses (3, 75%), allied health professionals (4, 100%), including dietitians, psychologists and speech pathologists, and GPs (4, 100%) and paediatricians (3, 75%) for care of their child.

#### 3.1.3 Phase Three—Prototype Development and Participant Feedback

PHPs (*n* 4) provided qualitative written feedback to the prototype clinical resource.

### 3.2 Clinical Needs Assessment

**FIGURE 2:**
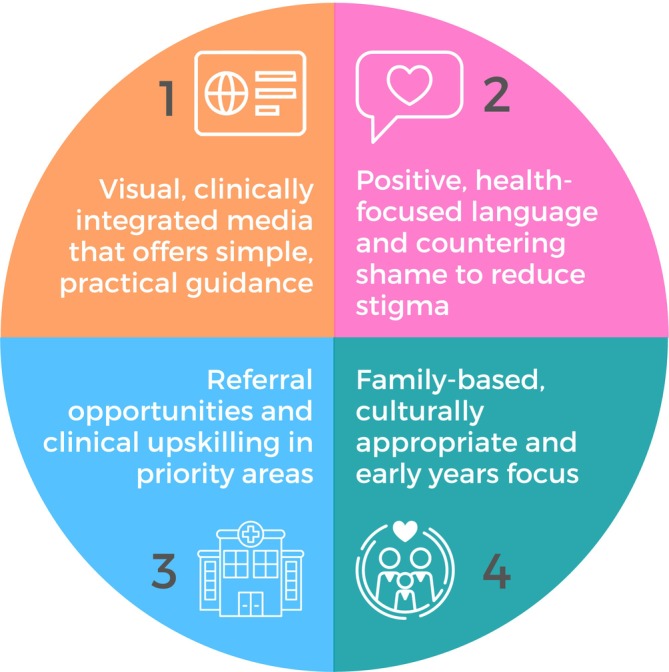
Final themes resulting from the clinical needs assessment for childhood obesity resources (*n* = 18 primary health care practitioners).

Rapid thematic analysis revealed PHPs identified unmet clinical resource needs across four themes (Figure 2). Visually engaging and technology‐driven media were the design cornerstones of clinical resources, with simplicity and practicality key for resource content. A gap was identified in how to use positive, health‐focused language to directly counter potential feelings of shame in children and families. Seamless integration with health service pathways was an unmet need; PHPs wanted resources to improve their knowledge of referral opportunities and receive clinical training in priority areas, such as prevention, risk identification and brief interventions. PHPs identified parent‐ and family‐based advice that is culturally adaptable to suit diverse cultures was necessary, especially during the early years (first 2000 days).

### 3.3 Participatory, User‐Centred Co‐Design

The online design workshop with PHPs (*n* 5) and caregivers (*n* 4) developed nine unique clinical resource solutions (one solution per participant) aligned to the workshop goal, requirements and scope (Table 2). Solutions ranged from traffic light systems to communicate health risk, clinical decision support systems to prompt routine height and weight measurement and analyse longitudinal trends in growth, and visual aids for identifying risk and protective factors for excess growth, as examples. Solutions that received the most participant votes and were agreed as priorities by participant and research team consensus in the workshop focused on: “Being BRAVE”—an acronym framework to help respectfully raise the topic of weight with families; “Too Much Love”—a strengths‐based framework to communicate to parents that providing excess food, often unhealthy, is providing too much love and could benefit from behaviour change.

### 3.4 Prototype Development: BRAVE Framework

**FIGURE 3:**
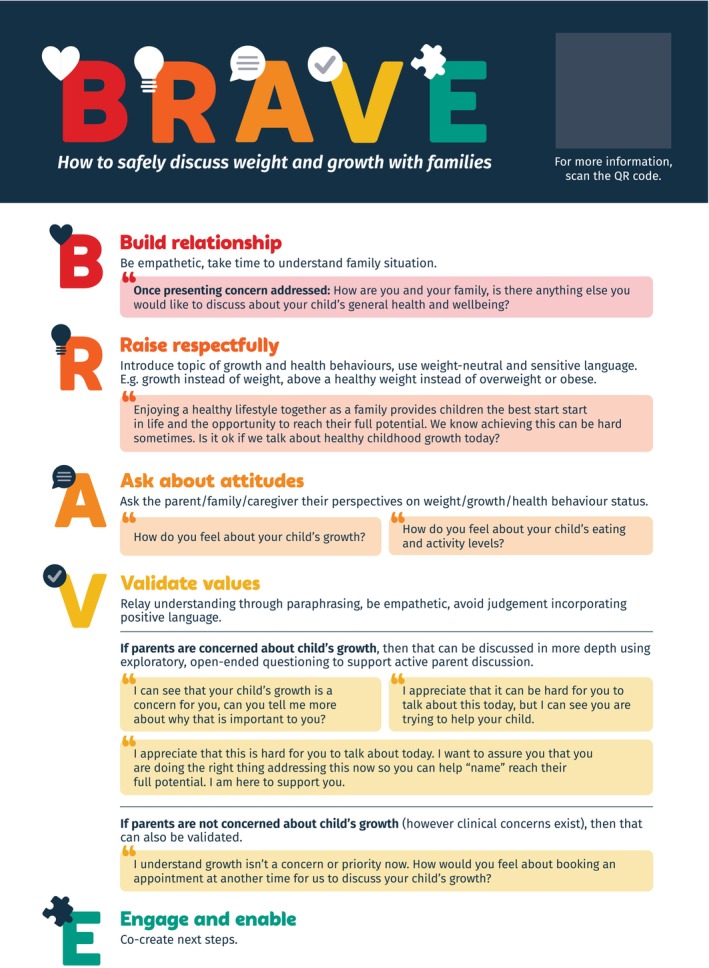
BRAVE framework to help primary healthcare practitioners raise the topic of weight and healthy growth in routine care with children and young people and their caregivers.

The research team reached consensus to develop the *BRAVE (Build relationships, Raise respectfully, Ask about attitudes, Validate values, Engage & enable)* framework (Figure 3) as a prototype due to its relevance to the research question, strength in addressing an evidence‐based clinical barrier and perceived ease of uptake and applicability to PHC. *BRAVE* is a framework to break down barriers to discussing childhood weight and growth in routine PHC. *BRAVE* is a targeted clinical resource designed to address the pervasive barrier of initiating a conversation about weight and healthy growth in practice, especially when weight is not the presenting concern. Its purpose is to improve PHP confidence and self‐efficacy to raise the topic of weight, and to encourage a non‐judgmental, safe, motivating and trusted environment for children and families. Changes to the *BRAVE* prototype were made based on PHP feedback, including providing sample phrases for PHPs to use at each stage, adding ‘enable’ to the ‘*Engage & enable*’ step, and suggesting to include a closed‐ended question of ‘is it okay if we talk about healthy childhood growth today?’, rather than an open‐ended question of ‘how would you like to discuss healthy childhood growth today?’ at the ‘*Raise respectfully*’ step. As a clinical prototype, *BRAVE* is ready for review by a larger population of PHPs and feasibility testing in practice with PHPs.

## 4 Discussion

### 4.1 Main Findings

This study engaged 18 multidisciplinary PHPs and four caregivers and (a) uncovered four priorities (Figure 2) to address persistent resource, education and training gaps that precisely target evidence‐based barriers to best‐practice prevention and management of childhood obesity; and (b) co‐designed nine unique solutions to help PHPs: talk about weight with families, deliver messages in a simple, practical and family‐centred way, and be easily integrated with current systems and clinical workflow. One solution *(BRAVE framework)* was developed as a clinical prototype for pilot implementation in practice; *BRAVE* is a structured, strengths‐based framework to assist PHPs in empowering discussions about weight and growth with families in practice.

### 4.2 Comparison to Previous Work

Clinical decision‐support tools and resources have the potential to augment clinician self‐efficacy to deliver evidence‐based decisions at the point‐of‐care [24]. Clinical resources for paediatric weight management have been developed in state‐based initiatives. NSW Health has developed free online clinical resources and training modules for health professionals via the *Healthy Kids for Professionals* website [11]. Many resources are instructional and target evidence‐based barriers (Figure 1) to management; for example, the BMI calculator provides an explanation of ‘healthy weight’, and a ‘conversation starter’ resource guides clinicians over the 4 A's framework (Assess, Advise, Assist, Arrange) to discuss healthy weight and growth [11]. Similarly, in QLD, the state's dedicated health promotion agency that is responsible for addressing obesity—Health and Wellbeing Queensland—has developed *Clinicians Hub*, an online portal with clinical models of care, referral pathways and tele‐mentoring programmes (ECHO Learning) to support clinicians, especially those working in rural and remote locations [12].

Whilst these resources exist and are useful and beneficial in PHC more generally, to improve rates of overweight/obesity management, practitioners require targeted assistance to help raise the topic of weight and healthy growth when it is not the presenting concern. Children and adults with obesity are presenting frequently to clinical services but are typically not being primarily treated for obesity [25]. In the most recent (2008) national estimate of general practice management of childhood overweight/obesity, the rate of GP management of childhood overweight/obesity was approximately one in 58 children, meaning only 1.7% of child patients with overweight/obesity were receiving treatment [7]. *BRAVE* (Figure 3) is an empowerment framework—for PHPs and families—to feel safe to discuss weight and healthy growth in routine care, especially when the presenting concern is not weight‐related. The *BRAVE* framework is simple, yet specific in its approach, and could potentially address substantive mechanistic barriers to managing childhood obesity in primary care, including low self‐efficacy, weight stigma and difficulty raising the topic of weight due to sensitivity [5] which is unlike other available resources. PHPs may adopt a *BRAVE* approach and its suggested structure when initially raising the topic to ground the conversation in a standardised framework that is routine for everyone, which could improve parent/caregiver acceptability [22]. It is designed to be used in conjunction with other resources, as on its own will not suffice to provide comprehensive overweight and obesity care. In this way, it is similar to the Ask, Advise, Help (AAH) model to support smoking cessation developed for use in Australia by primary healthcare nurses [26]. This model is a simple 3‐step process that is designed to support nurses to address smoking cessation with patients and links to other training and resources.

### 4.3 Implications for Practice

Until March 2022, there had been no universal strategic direction for addressing obesity in Australia. The *National Obesity Strategy* (2022–2032) was established due to decadal developments in obesity discourse and evidence and includes reference to improving integrated models of care, upskilling the healthcare workforce to prioritise clinical prevention, preventing weight stigma and supporting positive discussions about weight [2]. PHPs accurately reflected these evolutions in the present study; themes emerged relating to weight stigma and upskilling in prevention, risk identification and brief interventions to improve clinical practices.

Whilst a unified national strategy is an invigorating step, implementation remains its greatest challenge. Precision clinical resources, like those proposed in the current study, also require precision translation into existing referral pathways, electronic clinical systems and education and training programmes to maximise uptake. Implementation will be strengthened if aligned with health system reform—such as that already underway in many Australian states—that prioritises integrated and appropriately remunerated preventive health care [27]. One method of sustainable translation is integrating barriers‐focused training into multidisciplinary (medical, allied health, nursing) health professional education degrees and clinical placements to predict and prevent low confidence and self‐efficacy in priority areas, such as raising the topic and prevention in the first 2000 days. Translating the *BRAVE* framework into existing education and clinical systems could be improved if guided by the COM‐B model to maximise behaviour change [15]. This could include methods such as reminder screensavers as a reference point, integrating *BRAVE* into structured electronic medical record chart templates in all paediatric patient encounters, or using *BRAVE* during vaccination and nurse check‐up appointments.

### 4.4 Limitations

Our primary limitation is lack of cultural adaptation of the *BRAVE* framework; however, priority populations in Australia, such as Māori and Pacific Islander and Aboriginal and Torres Strait Islander peoples, require targeted engagement that is community‐driven and aligned with cultural practices. This was outside the scope of this study and will be prioritised in future research. Our sample was predominantly nursing and allied health PHPs and may not have adequately represented other PHPs, such as general practitioners, pharmacists and multicultural health workers. Caregivers of children aged 0–5 years were not represented in our sample. Additionally, the final *BRAVE* framework has not been externally validated with PHPs and caregivers outside the study participants PHPs who provided qualitative feedback.

## 5 Conclusions

Through a participatory co‐design research process, we co‐developed a new clinical resource (*BRAVE* framework) to help PHPs sensitively raise the topic of weight with children and families. This framework is underpinned by trust, empowerment and judgement‐free communication to improve the patient and clinician experience of weight management care that can potentially reduce weight stigma in health care. Future research can focus on external validation in a larger state or nationally representative cohort, pilot feasibility testing with PHPs and caregivers in a clinical setting to measure end‐user acceptability, cultural adaptation to priority populations and designing additional resources to meet the core unmet needs of PHPs for best practice prevention and management of childhood obesity.

## Ethics Statement

This research was approved by The University of Queensland Human Research Ethics Committee (2020002188).

## Conflicts of Interest

The authors declare no conflicts of interest.

## Supporting information


File S1.

File S2.

## Data Availability

The data that support the findings of this study are available on request from the corresponding author. The data are not publicly available due to privacy or ethical restrictions.
