# Supplementary material for: Raising the Topic: Clinical Needs Assessment and Co‐Design of Targeted Clinical Resources for Primary Healthcare Practitioners to Prevent and Manage Childhood Obesity
Source: Health Promot J Austr. 2025 Mar 13;36(2):e70033. doi: 10.1002/hpja.70033 (PMC11906268; doi:10.1002/hpja.70033)
Supplement: Supplementary file 2 — File S2. [file HPJA-36-0-s001.docx]

**Supplemental File 2**

**Title:** Raising the topic: Clinical needs assessment and co-design of targeted clinical resources for primary health care practitioners to prevent and manage childhood obesity

Table 3: Characteristics of primary healthcare professional (PHPs) participants (*n =* 18) – *clinical needs assessment*

| **Characteristic** | ***n* (%)** |
| --- | --- |
| **Clinical discipline** | 18 (100) |
| Nursing | **8 (44)** |
| *Registered Nurse* | 3 (17) |
| *Child Health Nurse* | 3 (17) |
| *Community Nurse* | 2 (11) |
| Allied Health | **7 (39)** |
| *Dietitian* | 3 (17) |
| *Physiotherapist* | 2 (11) |
| *Clinical Exercise Physiologist* | 2 (11) |
| Medical | **3 (17)** |
| *General Practitioner* | 3 (17) |
| **Health service area** | 13 (72) |
| *Major City* | 8 (62) |
| *Inner Regional* | 1 (8) |
| *Outer Regional* | 2 (15) |
| *Remote* | 0 (0) |
| *Very remote* | 2 (15) |
| **Years practising as a clinician** | 18 (100) |
| *<2 years* | 0 (0) |
| *2-5 years* | 1 (6) |
| *6-10 years* | 2 (11) |
| *11-20 years* | 6 (33) |
| *>20 years* | 9 (50) |

Table 4: Characteristics of design workshop participants (PHPs and caregivers) (*n*=9) – *participatory, user-centred co-design*

| **Characteristic** | ***n* (%)** |
| --- | --- |
| **Primary healthcare professionals** | 5 (100) |
| *Clinical discipline* |  |
| *Registered Nurse* | 1 (20) |
| *Child Health Nurse* | 1 (20) |
| *Physiotherapist* | 1 (20) |
| *General Practitioner* | 2 (20) |
| *Health service area* |  |
| *Major City* | 1 (20) |
| *Inner Regional* | 1 (20) |
| *Outer Regional* | 2 (40) |
| *Remote* | - |
| *Very remote* | 1 (20) |
| *Years practising as a clinician* |  |
| *<2 years* | - |
| *2-5 years* | - |
| *6-10 years* | 1 (20) |
| *11-20 years* | 1 (20) |
| *>20 years* | 3 (60) |
| **Caregivers** | 4 (100) |
| *Number of children* |  |
| *1* | 1 (33) |
| *2* | 2 (66) |
| *3* | 1 (33) |
| *Child age* | 8 (100) |
| *0-5 years* | 0 (0) |
| *6-12 years* | 4 (50) |
| *13-17 years* | 4 (50) |
| *PHPs previously seen* | 4 (100) |
| *Nursing* |  |
| *Registered Nurse* | 0 (0) |
| *Child Health Nurse* | 3 (75) |
| *Community Nurse* | 0 (0) |
| *Midwife* | 0 (0) |
| *Allied Health* |  |
| *Dietitian* | 2 (50) |
| *Physiotherapist* | 2 (50) |
| *Exercise Physiologist* | 1 (25) |
| *Psychologist* | 2 (50) |
| *Speech Pathologist* | 2 (50) |
| *Occupational Therapist* | 2 (50) |
| *Health Worker - First Nations* | 0 (0) |
| *Health Worker - CALD* | 0 (0) |
| *Medical* |  |
| *General Practitioner* | 4 (100) |
| *Specialist (e.g. Paediatrician)* | 3 (75) |

*PHPs, primary healthcare professionals; CALD, culturally and linguistically diverse*
